# Our approach to developing communities of practice to foster research capacities for the adult social care workforce

**DOI:** 10.3310/nihropenres.13461.2

**Published:** 2024-11-18

**Authors:** Ferhana Hashem, Wenjing Zhang, Rasa Mikelyte, Sweta Rajan-Rankin, Ecaterina Porumb, Olivia Trapp, Ann-Marie Towers

**Affiliations:** 1 Centre for Health Services Studies, University of Kent, Canterbury, England, CT2 7NF, UK; 2 School of Social Policy, Sociology and Social Research, University of Kent, Gillingham Building, Chatham Martime, England, ME4 4AG, UK; 3 Adult Social Care and Health Directorate, Kent County Council, Maidstone, England, ME4 1XQ, UK

**Keywords:** capacity building, social care research, communities of practice, shared learning

## Abstract

**Background:**

Efforts to build and foster adult social care research in England have historically encountered more challenges to its growth and expansion compared with health research, with a sector facing significant barriers in facilitating research activity due to a lack of resourcing, poor valuation or understanding of the profile of social care research. The landscape for supporting the social care workforce to use, engage in and undertake research in adult social care has been rather bleak, but in recent years there has been recognition of the need to foster a social care workforce research community. The National Institute for Health and Care Research in England have committed to investing in social care research capacity by funding six adult social care partnerships, with one based in Southeast England. Setting up Communities of Practice (COPs) offers a model to build a shared learning space to foster a social care research community. Process developing COPs: Three online networking events were held in the first year of the project to engage managers and practitioners from the local authority and from the wider adult social care sector, taking place in July and November 2021, and March 2022. Two COPs were identified, following an ordering and thematising process of feedback from the networking events, of: (a) Supporting people with complex needs throughout the lifespan, and (b) Enhancing, diversifying and sustaining the social care workforce. Whilst it would be premature to identify their long-term impacts, the COPs have provided a space for regular communication, knowledge sharing and networking between members.

**Conclusions:**

The COP framework offers a collaborative approach to initiating research from the grass-roots level in adult social care. This paper focuses on how the COP model offers great promise for knowledge-exchange providing a forum to generate and disseminate knowledge around social care in two COP domains.

## Introduction

Despite efforts to build and foster adult social care research in England, the pace at which applied health research has progressed under the National Institute for Health and Care Research (NIHR) in England has outstripped research in adult social care, which has historically encountered more challenges to its growth and expansion ^1^. The social care sector – including local authorities, third or private sector organisations – face significant barriers in facilitating research activity, compounded by lack of resourcing and poor valuation or understanding of the profile of research activity in the sector ^2^. The constraints on resources allowing social care practitioners and managers to engage in knowledge production or growing the evidence base has been severely hampered by successive disinvestments in the social care sector and reductions in local authority expenditure, which has rendered the social care workforce with far fewer opportunities for research capacity development. There are also disparities within adult social care in terms of career development and research capacity building alongside the existing professional discrepancies between employees within the sector. As part of renewing their registration annually, social workers have some avenues to pursue profession-related continuing professional development (CPD) ^3^, but those working in the voluntary and private sectors ( *e.g.* care workers, care managers) have had next to no such opportunities highlighting an unequal access to CPD. The landscape for supporting research in adult social care has been rather bleak, but in recent years there has been recognition of the need to foster a research community ^2, 4^. The 2022–23 House of Commons report on workforce recruitment and retention indicates an urgent need to invest in the human capital of the health and social care workforce in the UK ^5^. Since the early 2020s, the NIHR have committed to investing in social care research capacity building by funding six adult social care partnerships, one of which is based in Southeast England involving a consortium of organisations led by the local authority and the county’s largest higher education institute (HEI) ^1, 2^.

There are several issues that have historically inhibited the growth and development of social care research in England. In a permanently resource-strapped sector, the social care workforce in England has been thrust into a reactive mode of operation, with a practice-focus and low valuation on social care research. There has been a tenancy in health research to rely on evidence generated through generalisable, large-scale observational or controlled studies, which has reflected the greater level of research investment in this sector. Whereas social care research has valued evidence generated through ‘knowledge-based practice’ recognising that the application of wisdom of practitioners, and the lived experience of service users can be an equally warranted epistemological approach to formal research ^6^. In addition to the constraints on the adult social care sector to conduct research, people who draw on care and support are usually accessed via gatekeepers such as providers, commissioners or unpaid carers ( *e.g*. family/friend), and this means researchers have to navigate multiple levels of approval to engage directly with those who use adult social care, and due to the disparate nature of the sector it is difficult to facilitate or support research ^4, 7^.

The capacity to produce research in social care is vital to increase research literacy and ought to be part of professional education and training ^8^. In spite of a long-established consensus that local services and policies are better informed by research evidence, the backdrop of a sector facing a significant workforce crisis poses substantial challenges for organisations to enable social care practitioners and managers to attend conferences and seminars to exchange ideas and contribute to shared learning ^2^. In an environment beset by constraints on resources and time, a learning practice culture that moves away from professionally managed learning to humanistic and democratic learning offers a holistic approach that re-focuses the importance of the group’s skills, knowledge and experiences on the learning process ^9^. Communities of Practice (COPs), as described by Wenger *et al*. ^10^ provide an informal learning space for groups of people who share a concern, set of problems or passion about a topic, who wish to deepen their knowledge and expertise in this area by interacting with others on a regular basis. The opportunity to spend time to share information, insight and advice to solve a problem or ponder over common concerns offers a forum for building knowledge and learning together. Over consecutive exchange sessions, the group may develop a unique perspective on a body of common knowledge, practices and approaches ^10^.

### Valuing research capacities in adult social care

Without access to quality research and evidence, social care practitioners can be ill-equipped to up-skill or be aware of cutting-edge innovations, and more crucially, be able to critically evaluate evidence for best practice ^11^. Robust evidence is also needed to inform the public and it is essential that people who draw on care and support and their carers have the best evidence available. At the same time, the voices of people who draw on care, support and help from carers, are an integral part of social-care related research and their inclusion in research can also ‘add value’ to contribute to the evidence base, maximising the impact of research in terms of support and interventions in the sector ^12^. Lastly, evidence is needed to inform people who draw on care and support along with carer communities, and their direct involvement in contributing to the evidence base is fundamental in developing services that meet their needs ^8^.

### Difference in research funding between care sectors

Dixon and colleagues have noted in their scoping review on the commissioning of social care research, that relative to health research, available budgets for social care research have tended to be much smaller ^1^. In 2022, the NIHR reported spending £90 million on social care studies over the last three years ^13^. Despite this being a notable increase, NIHR has spent more than £250 million a year on all research; therefore social care research spend still remains a fraction of the overall budget ^14^. The recent tranche of funding committed to invest in public health research under the NIHR’s Health Determinants Research Collaborations (HDRCs) was £50 million awarded to 13 local authorities ^15^, yet, the amount of funding for the six adult social care partnerships was staggeringly lower, totalling just over £8 million under NIHR’s Health and Social Care Delivery Research Programme (HSDR). Given the social care research footprint is a rather tangled web of blurred boundaries, many areas of social care research remain unaddressed alongside the outstanding research gaps in the evidence base ^1^.

### Issues with research capacity in the social care workforce

Prior to the establishment of the NIHR in 2006, it was recognised there was a notable gap in the range and volume of social care research, and recommendations were aimed at increasing the evidence base for social care practice ^8^. However, implementing strategies targeted at encouraging front-line staff to develop their research skills and knowledge have not been prioritised. Almost a decade later, solutions to increase social care research capacity remained stagnant. A 2015 survey of 70 English local authorities (317 in total in England) ^16^ on research capacity, knowledge, and skills use in councils with adult social care responsibilities, found that local authorities received little support or resources for investing in research capacity development, with some local authorities not believing they needed to facilitate training to enable staff to acquire and develop their research skills ^2^. Furthermore, one of the key barriers to any significant investment in research had been the austerity policies introduced by the Government between 2011 to 2015, which substantially reduced local authority expenditure by 25%. The consequences of which were felt particularly to staff research development initiatives which were seen as ‘non-essential’, and until recently, efforts to foster research capacity in social care had been pushed to the fringes ^2, 17^.

## Framework for creating a social care research community through Communities of Practice (COPs)

In this section, we discuss how research activity in social care is being fostered through establishing research-focused Communities of Practice (COPs) ^10, 18– 20^, a shared learning model aimed at situating learning and building a research culture to deepen knowledge and lay the foundations for an active social care research community ^21^. It reports upon the consultation exercise and development of two COPs on (a) Supporting people with complex needs throughout the lifespan and (b) Enhancing, diversifying and sustaining the social care workforce. The paper also discusses the mechanisms in place to support them, including a cloud-based collaboration platform providing a scaffolding for information and resource exchange. With the COPs currently in their early phase of growth since their launch in June 2022, the focus is on the initial stages of their development and how a COP framework offers the potential for building sustainable research capacity development in adult social care. Despite the coronavirus (COVID-19) pandemic originally posing a challenge to their set-up, development and launch, the partnership based in Southeast England has also proven that moving the COPs to an entirely online process offers great promise to sustain an online community of COPs for knowledge exchange and shared learning.

COPs are characterised by a unique combination of three fundamental elements and are important as a model for research capacity building ^21, 22^: a domain of knowledge, which defines a set of issues; a community of people who are concerned about this domain; and shared practice – that they are developing to be effective in their domain ^10^. Once they function in unison, these three elements comprise of a community of practice, a functioning social structure organically taking responsibility for developing and sharing knowledge. Early buy-in is also vital to the success of COPs and in order to ensure they thrive, a life-cycle framework is essential to underpin their development. Cambridge *et al*. ^23^ define these stages as: Inquire (identifying audience and purpose), Design (defining activities and roles to support goals), Prototype (pilot the community with stakeholders), Launch (roll out to a broader audience) and Grow (engage members in collaborative learning and sharing activities). This framework has been integral to fostering the COP partnership’s adult social care capacity building initiative, funded by NIHR’s programme Health and Social Care Delivery Research (HSDR; NIHR131373). Below we have outlined how we have used these stages to develop our partnership’s COPs. Ethical approval was not required to set up the COPs, which was indicated in accordance with the Health Research Authority’s decision tool (see ethical review statement below and data availability statement).

## Collaboration process developing the COPs

### Creating and establishing the COPs

Developing the COPs was prefaced by three online pre-engagement activities aimed at growing and developing the learning partnerships, which required a deeper mutual learning stage to help generate new ideas and research hypotheses involving engagement with social care practitioners, managers, people using services and their unpaid carers, local authority and sector stakeholders. Three large networking events were held in the first year of the project to engage managers and practitioners from the local authority and from the wider adult social care sector. These took place in July and November 2021, with a last event in March 2022. The purpose of these online events was to (i) identify and discuss priority areas in which to focus on in adult social care research in the county, and (ii) introduce communities of practice and focus on how they can support best practice and practice-orientated research.

***Inquire***

**Figure 1.**
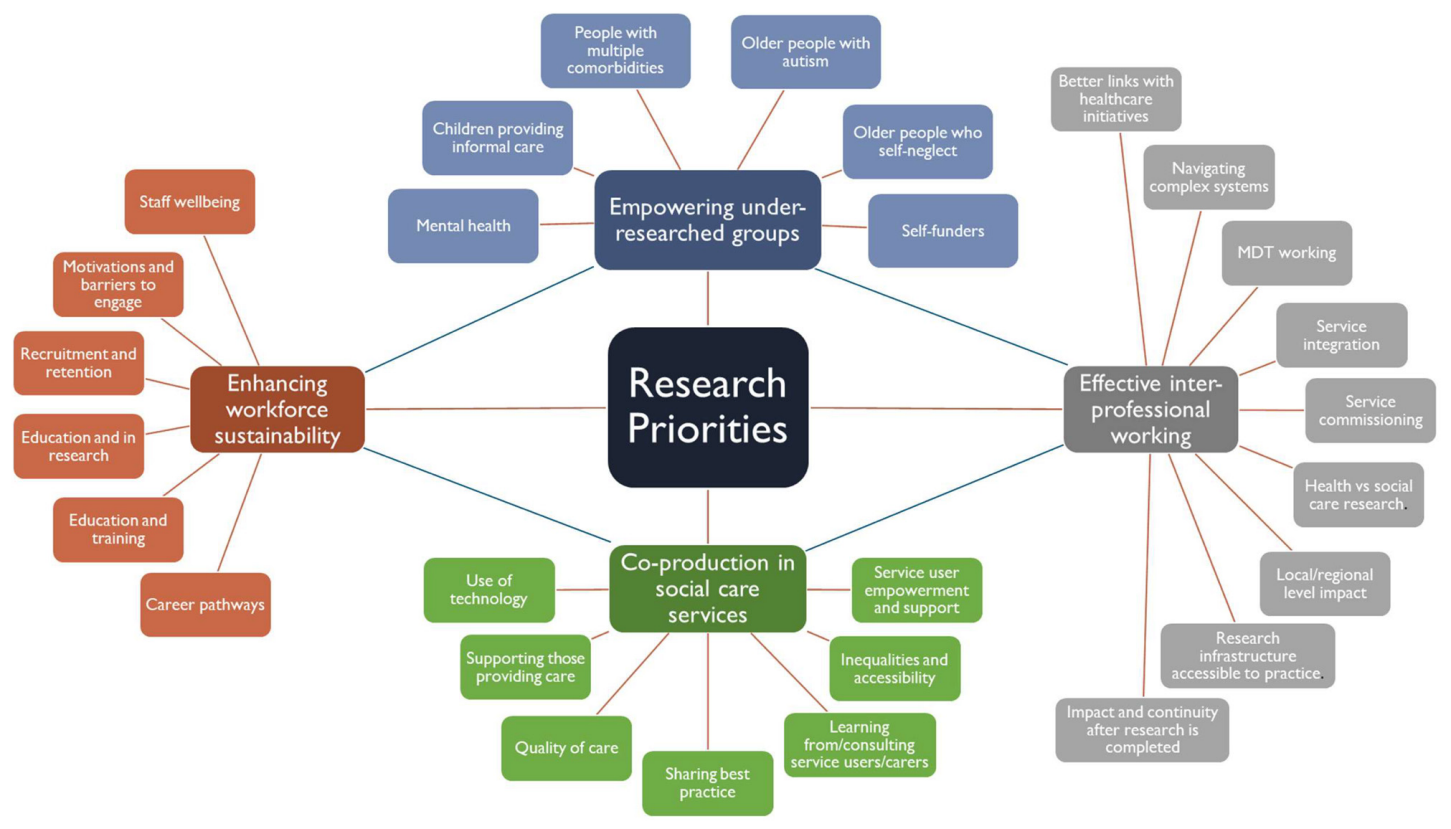
Research priority areas identified at Kent Research Partnership’s first networking event.

The first online networking event served to brainstorm and capture key research ideas and talk about the ways in which COPs can contribute to shared group learning. This was attended by 113 people from diverse backgrounds including experts by experience (people who draw on care and support and their informal carers), service providers, researchers and care commissioners. Stakeholders were invited by the innovation delivery team, based in the local authority’s adult social care and health department, a collaborative social enterprise supporting local care providers, a national not-for-profit forum promoting quality care, a UK membership body for homecare providers, and through snowballing by the project’s chief and co-investigators. As a networking exercise, to enable full engagement of all people, stakeholders were not purposively identified, and to this end, people were not asked to indicate if they were attending on behalf of a specific organisation or sector. Seven facilitated online breakout sessions were conducted and led by members of the project team. Facilitator briefing notes were compiled and the sessions were video-recorded with the permission of contributors. We produced instantaneous Word Clouds based on the discussion notes from the seven breakout discussion groups. Each group had two facilitators who took notes and saved them to a shared document. This allowed one qualitative researcher to produce the Word Clouds during the event and showcase them to the audience at the end. For example, workforce, training, wellbeing, dementia were some key words from the discussions around *“what are the key research priorities in adult social care in [county name]”*; with time, practitioners and user engagement frequently mentioned when discussing *“how to support practice-focused research in [local authority] adult social care in [county name]”*. Following the first event, the ideas from the breakout sessions were revisited and thematically coded using NVivo, when we generated additional Word Clouds on the discussion topics (i.e., "What are the key research priorities in adult social care in Kent?" and "How to support practice-focused research in adult social care in Kent?"). The Word Clouds feature in the top 70 frequently mentioned words for each question, excluding common terms like “research”, "social care," and "the". (see Figure 1 for thematic coding framework) with four research priority areas identified: (a) inter-professional working, (b) empowering under-researched groups ( *e.g.* self-funders, self-neglect, older people), (c) enhancing workforce sustainability, and (d) co-producing social care services.

We invited stakeholders who had previously attended the first networking event to a second online networking event, taking place in November 2021, which was promoted widely via our local networks including the local authority, an independent body supporting local care providers, NIHR Applied Research Collaboration [withheld] partnership and the HEI. We had 117 registered attendees overall, again from diverse backgrounds including experts by experience, service providers, researchers and commissioners. For the second event, the same approach was enlisted for reaching out and involving people, as described for the first networking event. The purpose of this second event was to (i) present and discuss the four identified research priorities and (ii) develop the communities of practice with regard to organising and facilitating the sessions. The attendees had the opportunity to give their initial responses to the identified research priorities and indicate how the COPs could be best facilitated through two separate breakout sessions. The first online breakout session involved an open discussion with the facilitators asking attendees *‘what do you think of these research priorities?”*, with a video recording of discussions being undertaken with the permission of contributors.

**Figure 2.**
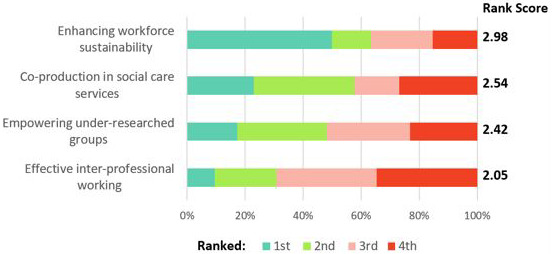
Priority area ranking exercise results identified at second networking event (N=52).

Following the first breakout session, attendees were asked to indicate their responses to the research priorities through a ranking exercise, which was set up using an online survey tool called Qualtrics and the weblink for responses was made available at a specific time point during the breakout session (see Figure 2). Responses were received from 52 attendees, with results indicating that enhancing workforce sustainability was ranked highest overall, followed by co-production in social care services and empowering under-researched groups being closely ranked as second and third priority areas respectively, and effective inter-professional working designated least overall priority. The results were not representative of all respondents’ views but provided indicative scores of the shared priority setting exercise.

Before the second online breakout session commenced, the live results of the research priority ranking exercise were shared with the attendees, followed by a short discussion on the proposed aims and scope of the COPs with an opportunity for attendees to ask questions. In the second breakout session, attendees were asked *‘how can we translate the research priorities identified into Communities of Practice?’* The discussions were video recorded with the permission of contributors.

***Design***

**Figure 3.**
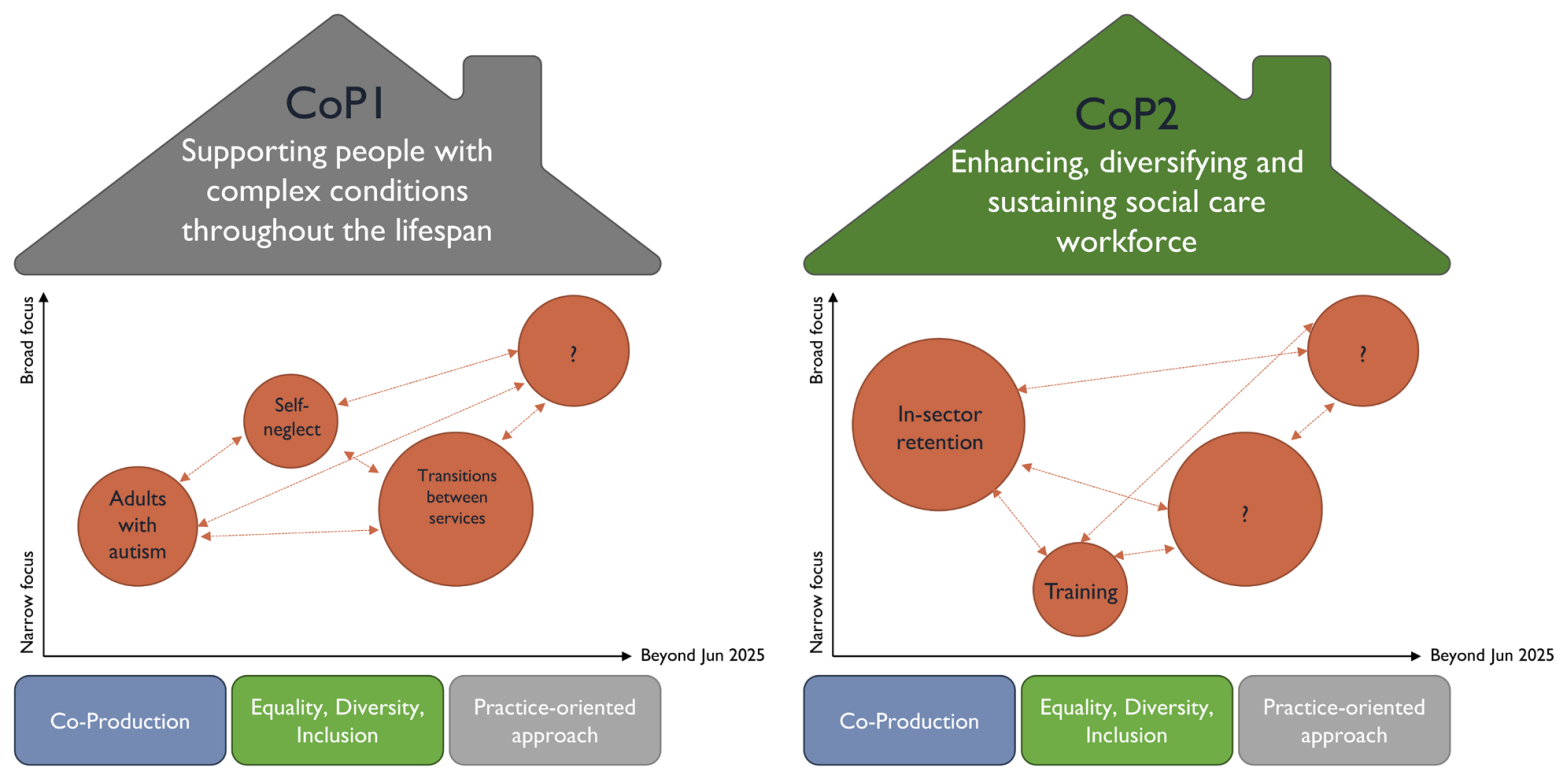
Communities of Practice ‘Houses’ outlining domains.

After the second networking event, an ordering and thematising process took place to take on board feedback from attendees to help define COP activities and roles to support its goals. The recorded discussions and responses from the second breakout session were organised into themes and helped to identify key issues of concern or domains for the COPs. The themes were arranged under two broad ‘rooves’ of the community (house) as shown in Figure 3 and were titled: (a) Supporting people with complex needs throughout the lifespan (shortened to ‘Complex Needs’), and (b) Enhancing, diversifying and sustaining the social care workforce (shortened to ‘Workforce’). An inductive-deductive approached was used in the thematising process, with inductive coding themes emerging from the facilitated discussions, and deductive coding themes being derived from the coding framework developed after the first networking event ^24^. Three shared principles were identified as ‘foundation stones’ for each COP: Co-Production; Equality, Diversity, Inclusion and Intersectionality, and Practice-Oriented Approach, which were underpinning themes that were identified from the initial ordering and thematising exercise and intersected across both COPs (see Figure 3).

***Prototype***

A third online networking event occurred in March 2022 with the primary purpose to initiate the COPs around the two COP domains, with the express purpose to allow attendees to ‘try-out’ a COP through a mini session of both groups. A total of 83 people attended (experts by experience, service providers, practitioners, researchers and commissioners). For the third event, the same approach was enlisted for reaching out and involving people, as described for the first and second networking events. We brought together and launched the COPs at this third event when the houses were presented and a Google Jamboard (a digital whiteboard suitable for online collaboration) was used in each breakout room to identify more ideas into each of the houses. A variety of topics were discussed under each themed domain. Examples are represented as evolving bubbles in Figure 3, with distinct sizes (dependant on the scope of the question and number of COP members who were interested in it), different levels of focus (narrow/broad) and mapped against different timelines. The latter acknowledges that COP members would not focus on all interest areas at the same time, whilst also recognising the interrelation of specific topics within a COP through connections.

Along with the two online break-out sessions enabling attendees to discuss the topics and try-out the COPs (with a record of discussions being undertaken using the same approach as the first and second events), they were also asked to participate in a short-survey to record which domain attendees shared a common area of interest in. Two people selected ‘other’ as an option, one stating they would like a community of practice focusing on *‘how to promote research in social work?’*, which was deemed within the scope of the Workforce COP. The second person suggested a COP on the *‘long term impact of long COVID especially in young people and its effects on mental as well as physical health; likely future implication for social care’*, which was assigned to the Complex Needs COP. The information collected at this last networking event showed that interest belonging to either one of the COPs was evenly split, therefore the COP domains seemed to have salience and suitability with the attendees to enable the final launching for the COPs to take place in the forthcoming months.

***Launch and grow***

The COPs were launched online in June 2022, with an organised schedule of dates planned across the first year occurring on a monthly basis. Due to the COVID-19 pandemic, all of the COP sessions were organised online and have continued to function online, as this has offered the flexibility of attending in and around work or care commitments. We also consulted COP members on their preferences of time of meeting, and jointly decided to hold these meetings in the middle of the day to enable practitioners to attend during lunch times. The format of each COP has focused around a presentation by one or more speakers on a topic relevant to the domain. Talks for the ‘Complex Needs’ COP have included: choice, control and direct payments, work transitions between children’s and adult’s services, supporting moves of older people between care settings, inclusive social care practice and commissioning, and technology use in adult social care. For the ‘Workforce’ COP, topics have included: recruitments and retention issues ( *e.g.* what we can do to make working in social care more attractive for young people), workforce concerns from a provider/funding perspective, spotlight on research embedded in care homes, workforce data, career development pathways, support for care workers, and experiences and expectations sought from social care.

### Engagement and facilitation

The importance of engagement and facilitation of the COPs are critical to their success (Padilla *et al.*, 2021). Yet, the constraints on sector staff time have required a rethink of how the COPs would traditionally have been organised and facilitated. The partnership’s team members based at the HEI and local authority have acted as convenors, providing a space for both the organisation and facilitation of discussions across the two COPs. This has helped maintain regular effective communication and continuity, keeping channels for shared learning open and making sure presentations address the needs of COP members ^25^. Membership of the ‘Complex Needs’ and ‘Workforce’ COPs have reached up to ~115 to ~118 respectively, and some people are members of both, whilst others only belong to one. The decision to hold the COPs online was initially a consequence of the COVID-19 pandemic, in place to limit physical interactions, but has allowed for the involvement of a broader range of stakeholders and COP members. The virtual platform has helped facilitate accessibility of the COPs to members using communication technology, carers and cared for people based at home and for a time-poor sector, namely the social care workforce. Importantly, successful use of virtual platforms for the COP meetings as well as for discussions and document share in between the meeting, hinged upon bespoke training sessions on using Zoom, MS Teams and Glasscubes platforms (five sessions in total), as well as in one case a researcher visiting an expert by experience in their home to assist with digital set up and discuss optimal ways of using communication devices ( *e.g.* speaking aids and text readers). Outside of COP meetings, researchers also worked with the experts by experience group (all of whom attend one or both COPs) by seeking routine feedback and amending how COPs are run accordingly. For example, additional IT training sessions, diversifying ways in which COP members are invited to the meetings and varying days of the week when COP meetings take place were all introduced as a result of this.

During COP meetings, the facilitator role has ensured that members are given the chance to speak, supported by an additional team member responsible for checking the web-chat function in order to address any questions raised by COP members ^26^. Discussions for each COP have involved working together to identify evidence gaps, with the view to plan research projects that are relevant to the community’s goals and interests. The opportunity for COP members to develop a research idea independently into a social care research project has been set in motion through the availability of research and training fellowships (up to £90,000 each), which COP members can apply to. These fellowships aim to bridge the training and skills gap for social care practitioners interested in research, thereby both fostering and supporting research capacity development from within the group (see Kent Research Partnership fellowship information video). At the time of writing, 20 sessions for each of the COPs have taken place which have enabled COP members to share knowledge and experiences fully engaging in a rich dialogue about key concerns and challenges, airing issues and brainstorming ways forward, and providing insight of ways to support best practice and practice-orientated research.

### Enrichment and online resources for supporting collaboration

Using cloud-based collaboration platforms to support online research communities provides a mechanism to encourage working together. Enabling the storing and sharing of information outside of an organisation’s firewall, in a space that is safe and accessible for all users based within or external to an organisation has encouraged ongoing engagement with the COPs. Each COP has a dedicated workspace enabling COP members who have joined to share thoughts, post interesting information and announce relevant opportunities. Supporting accessibility and increased functionality for COP members is paramount, and introductory training sessions are vital to induct new users to engage with the online platform. In order to facilitate buy-in and grow membership of the COPs, videos have been created explaining what the role and function of the COPs are to encourage new members to join ^27^. Creating a COP handbook has provided all members a focus on the group’s guiding principles, rules of conduct, membership entitlements, operationalising the group according to ethical principles, as well as outlining the logistics of how to join online. Using an online platform has shown how an online space offers both COP facilitators and members a vehicle to communicate regularly between COP meetings, support direct discussions, and undertake knowledge sharing and networking between our COP members and ultimately democratises knowledge-exchange for COP members, involving all corners of the sector experts by experience and social care practitioners and managers.

## Discussion

This paper offers insight into the ways in which group sharing and learning can empower the social care workforce to engage with and grow their research skills – essential to a sector which has historically seen an under-investment in research-focused education and training. The successes of using the COP model offers great promise as a framework for knowledge exchange providing a forum helping to generate and disseminate knowledge around social care, whilst remaining on topic within the COP’s two domains. Padilla *et al*. ^27^ similarly report upon the design, development and implementation of using the COP model as a framework for guiding clinical practice, identifying issues and building a sustainable relationship between an academic-practice partnership for nurse practitioner students at the Duke University School of Nursing. Padilla and colleagues note how the COP proved to be a success not only in expanding knowledge-exchange activity, but also strengthened the relationship between the nursing school and clinical practice settings ^27^.

The COP sessions facilitated so far have provided social care practitioners and managers with shared learning through research and best practice, supported by the partnership’s other capacity strengthening workstreams. These activities include: Research and Training Fellowships; ‘Two-sides of a Coin’ training sessions, linking practice-informed research and research-informed practice; and research capacity building support through ‘embedded-researchers’, following the Researcher in Residence model ^28^. Thus the COPs offer a platform to embed and consolidate the wider capacity-building initiatives available in the partnership ^25^. The COPs’ collaborative learning platform Glasscubes provides members an avenue for knowledge mobilisation, and there is the prospect to expand its membership outside of the immediate project and region, further facilitating access to resources, training and collective learning, as part of a new initiative beyond the life-cycle of the project ^25^.

Applying a proactive and responsive approach to supporting the COPs has proven to be effective in facilitating the discussions and retaining and expanding COP members. This includes our instantaneous summaries and follow-up actions after each networking and COP meeting, reporting back to the group about how their feedback has shaped the development of the COPs, and enabling ongoing and member-driven discussions in-between monthly COP meetings via the online platform. This approach has also helped to engage with and benefit experts by experience, who have actively participated in meetings and discussions on the online platform. Continuing to respond to the needs of each COP has enriched our understanding of what has worked and has helped to identify some of the key challenges.

The COPs are still in their infancy and continue to evolve organically ^25^. Yet, as the COPs gradually mature, it is essential to ascertain their impacts in addressing the challenges of social care capacity development. Evaluating the COPs will be central to understanding if a change in culture or practice has taken place, which can be notoriously difficult to measure and attribute to a specific intervention. Drawing from Cooke’s (2005) framework for evaluation based upon six principles of capacity building ^29^, the evaluation will involve four key elements: (i) description of each COP, its members, goals and objectives and a report of how well it met those objectives; (ii) experiential and reflective feedback from members about being involved in the COP, including view of workshops and training opportunities; (iii) summary of the facilitation and barriers to successful implementation, lessons learned and recommendations for the future; and (iv) assessment of members’ research confidence, skills and engagement, including whether they were successful in accessing fellowships or being involved in project applications. With the support of the researchers, it is expected that some COP members, with their newly acquired research skills, will be involved in their own COP evaluation and with support, provide a draft summary report for the partnership ^30^.

Some of the challenges encountered have focused on logistical issues encountered. Although the COPs have been active since the summer of 2022, the period leading up to their development from July 2021 until their kick-off in June 2022 took eleven months, which may have lead people who had attended those original events to lose interest and not join a COP. The delay in their launch can in part be attributed to the ongoing COVID-19 pandemic, which saw unparalleled pressures on the social care workforce, but nevertheless impeded progress on their eventual launch. In addition, as a consequence of the pandemic, the original plan was to host the COPs in person, but was moved online to maintain social distancing and encourage people who were shielding to have the opportunity to attend. We appreciate that on the one hand, this may have impacted on members’ shared learning experience who may have been more willing to contribute if the sessions were in person. Yet, on the other hand, COP members who may have been time-limited or less able to travel have nevertheless been more willing to join the sessions as these have been held online.

Logistical issues aside, some of the more critical challenges concern engagement from marginalised groups, including the need to represent the heterogeneity of the adult social care workforce, and accommodating all opinions amongst mixed stakeholder groups. The sustainability of the COPs succeeding in a shrinking resource-strapped sector is an underlying concern, and the end of the partnership will involve a withdrawal of the researchers supporting and facilitating the COPs, unless further funding to support these roles can be acquired. Understanding and securing the financial and operational mechanisms to deliver change through process, people and intervention will be paramount in the remaining two years of the project.

## Conclusions

In a climate beset by challenges to social care practitioners and managers to engage in knowledge-production and contributing to the evidence base to inform best practice, ongoing efforts to actively expand shared learning and knowledge mobilisation require a move away from professionally managed learning to democratic and holistic learning, re-focusing on the group’s skills, knowledge and experiences. The COP model has offered a pioneering and novel approach to building research from the grass-roots, based upon a shared research priority setting exercise which helped conceptualise key issues of concern, leading to the identification of the two COPs around the domains of ‘Complex Needs’ and ‘Workforce’. The three networking events were also pivotal in generating and recruiting membership to the COPs, as attendees were encouraged to feedback on the topics informing the subsequent formation of the two COPs, which has assisted with gaining early buy-in for their eventual launch. With the COPs in the initial stage of maturity, it would be a little premature to identify their impacts, but if we take engagement and attendance as an indicator of their value, it would appear there is an express need for creating and building research in the social care community. The evaluation strategies described to ascertain their impacts will bear fruit at the end of the project helping to understand their contribution and significance. Our experiences nevertheless describe a process in which an academic-public partnership can enrich research skills and build knowledge outside of traditional approaches.

## Consent and ethics

It has been important that the shared priority setting methods were chosen and applied ethically, which have underpinned the domains of our COPs. Nevertheless, we understand that priority setting partnerships do not usually fall under the remit of the UK’s Health Research Authority (HRA) ethical approval processes ^31^.

To ascertain whether this work has required ethical approval, FH submitted information on Work Stream 1 of the project using the HRA decision tool, as recommended under the James Lind Alliance’s Consent and Ethics guidance ^32^, which confirmed the study would not be considered research by the HRA. Evidence of the HRA decision is available as extended data (see data availability statement).

Ethical approval has been granted from the School Research Committee, by the Division for the Study of Law, Society and Social Justice at the University of Kent on 10/10/2022 (application reference: 0708). Approval has been granted for Work Package 1 for evaluating the impact of the COPs including permission to audio record interviews with members of the COPs on their experience of and impact of being a member of the COPs.

## Data Availability

No data are associated with this article. Figshare: Kent Research Partnership - HRA Decision making tool outcome, https://doi.org/10.6084/m9.figshare.23926176.v1 ^33^. This project contains the following extended data: HRA decision (the document shows the outcome of the decision tool, as not requiring UK ethical review) Data are available under the terms of the Creative Commons Attribution 4.0 International license (CC-BY 4.0).
