# Supplementary material for: Our approach to developing communities of practice to foster research capacities for the adult social care workforce
Source: NIHR Open Res. 2024 Nov 18;3:43. Originally published 2023 Aug 24. [Version 2] doi: 10.3310/nihropenres.13461.2 (PMC10593316; doi:10.3310/nihropenres.13461.2)
Supplement: Peer review history

# Reviewer report: Reviewer response for version 2

**DOI:** 10.3310/nihropenres.14837.r33833

**Published:** 2024-12-12

**Authors:** Louise Brown

**Affiliations:** 1 University of Bath, Bath, England, UK

The paper addresses the problem of growing the evidence base, developing the research capacity and engaging in knowledge production against a back drop of disinvestment in social care. It begins by setting out the context of social care and the challenges associated with facilitating research activity in this field. The abstract clearly presents the context of the work and provides a short overview of the paper.  It highlights the need for communities of practice, the process of establishing one and explores the potential of this model to foster a social care research community. It concludes the COP offers great promise in initiating research from grass-roots level in adult social care.

  The introduction provides a more detailed overview of the context and challenges discussing not least the lack of opportunity for CPD and the ever present problem of retention and recruitment into the sector. Within the introduction the paper provides important data on the lack of funding for adult social care research and discusses research capacity impacted by austerity measures.

 The paper reports back on one COP established in the Southeast of England which sought to address two topics, supporting people with complex needs and issues relating to workforce development. The stages of developing a COP are later set out in detail. I would have liked to see some detail on how the project as a whole emerged, who drove it and whether it was initiated by a local authority or a HEI? Who applied for the funding and who set up the pre-engagement activities? I think this is important when you are thinking about securing longer-term buy-in. Was the COP partnership developed from a standing start or is it important to already have some degree of partnership happening already?

 The paper details the process of the COP very clearly.  I was not entirely sure I grasped what the status was of the ‘Foundation stones’ for each COP? I understand how they were derived but what is their relationship to the overall exercise?

 In the sections on ‘Launch and grow’ and ‘Engagement and facilitation’ there is much detail about the process, who attended and how the events were managed. Yet I couldn’t quite understand what was being aimed for here? Were participants being trained in how to find and use research or being given research to use in their practice/roles/lives? it says speakers presented topics relevant to the identified domains. That makes sense but what were they presenting? Were they presenting up to-date research findings on that topic or was the purpose to get the group to develop a research idea into a project? It does state the COP enabled members to share knowledge and experience and engage in a dialogue about concerns and challenges which led to insight into how to support practice-orientated research – was that the overarching purpose? I think the paper would benefit from greater clarity of the purpose of the COP, what did it hope to achieve? Is it ultimately about growing research skills or more as in the discussion it talks about COPs being a means of disseminating knowledge around social care too. Or is it a means for research priority setting as stated in the conclusion?

***Some detailed points to consider:***

 -In the introduction one sentence mentions a ‘tenancy in health research…’ should it read ‘tendency’?

 -Reference number 8 (Marsh & Fisher, 2005) is quite old, are there anymore up to-date references that would support this comment?

 It might be good if in the discussion the authors could identify what are the lessons learnt more generally about COPs? I was a little confused in the discussion as to what the other workstreams were and how COPs fitted in? These are introduced to the reader very late on and could be mentioned in the introduction as context. I was interested in the role of HEIs and whether COPS rely upon HEIs to initiate them? Are there examples where they have been driven by a LA?

 The value or potential impact of the paper lies in its ability (through COPs) to try and increase research literacy amongst practitioners. It sets out what a COP is early on. It perhaps needs to be clear that the argument here is that COPs offer an inclusive means through which to improve research literacy but are not necessarily going to address the bigger challenges outlined in the abstract, ie the disinvestment in social care research. The paper is very positive in its promotion of COPs. I wonder whether it should flag that these appear to offer potential but cannot in and of themselves address the bigger issues and challenges created by austerity policies. They are one stage of a long and complex process required to generate an evidence base for adult social care, it would help if the paper was really clear as to what stage of the process they aim to contribute to.

 In relation to the overarching questions.

 Is the work clearly and accurately presented and does it cite the current literature? Yes, I would assess the paper as having met this standard. Some of the references could perhaps be updated if literature exists.

 Is the study design appropriate and does the work have academic merit? I thought the design was appropriate and had academic merit.

 Are sufficient details of methods and analysis provided to allow replication by others? I have identified that the paper might benefit from further clarity around the purpose of COPs and what they are able to deliver (and not).

 If applicable, is the statistical analysis and its interpretation appropriate? N/A

 Are all the source data underlying the results available to ensure full reproducibility? Yes.

 Are the conclusions drawn adequately supported by the results? Yes

Is the work clearly and accurately presented and does it cite the current literature?

Yes

If applicable, is the statistical analysis and its interpretation appropriate?

Not applicable

Are all the source data underlying the results available to ensure full reproducibility?

No source data required

Is the study design appropriate and is the work technically sound?

Yes

Are the conclusions drawn adequately supported by the results?

Yes

Are sufficient details of methods and analysis provided to allow replication by others?

Partly

Reviewer Expertise:

Undertaking research in adult social care. Co-production.

I confirm that I have read this submission and believe that I have an appropriate level of expertise to confirm that it is of an acceptable scientific standard.

# Reviewer report: Reviewer response for version 2

**DOI:** 10.3310/nihropenres.14837.r33678

**Published:** 2024-12-11

**Authors:** Kathryn Almack

**Affiliations:** 1 University of Hertfordshire, Hertfordshire, UK

Thank you for your revisions and responses to the reviewers comments. With reference to the question of identifying the background and breakdown of the stakeholders involved -  thank you for the rationale (do you now mention this in the article?), which I accept is reasonable and understandable. At the same time, it may be worth considering if, in signing up for an event, it may not be too onerous to attendees affiliation (e.g. Local Authority; Voluntary sector organisation) including an  option as well to state (If no affiliation) that they were attending as an interested individual.

Is the work clearly and accurately presented and does it cite the current literature?

Yes

If applicable, is the statistical analysis and its interpretation appropriate?

Not applicable

Are all the source data underlying the results available to ensure full reproducibility?

No source data required

Is the study design appropriate and is the work technically sound?

Yes

Are the conclusions drawn adequately supported by the results?

Yes

Are sufficient details of methods and analysis provided to allow replication by others?

Yes

Reviewer Expertise:

Social care research; qualitiave research; communities of practice

I confirm that I have read this submission and believe that I have an appropriate level of expertise to confirm that it is of an acceptable scientific standard.

# Reviewer report: Reviewer response for version 1

**DOI:** 10.3310/nihropenres.14603.r30319

**Published:** 2023-10-05

**Authors:** Agnes Turnpenny

**Affiliations:** 1 Oxford Brookes University, Oxford, UK

First of all, I would like to congratulate the project team/authors for their work and achievements on developing and fostering communities of practice. This article provides a useful account of the approach and activities and will be useful for other organisations looking to set up their own communities of practice. 

 I have some relatively minor comments that the authors might wish to consider:

- The introduction (the three "why" subheadings) feels a bit long and repetitive. Some of the information and points seem to be presented multiple times. I wonder if there is a way to make this more succinct.
- Description of the collaboration process: I wonder if this could be more clearly organised around the lifecycle framework presented at the start of the section. Currently the first three stages and the last two stages are presented together. Breaking the information down would improve the clarity of the process description. 
- Glasscubes: whilst it is important and useful to include information about specific tools, I wonder if this section could be made more generic and focus on the requirements of the online collaboration rather than what Glasscubes can do. I imagine other platforms are available and some communities of practice might have to find platforms that are free to use. There is also a contrast with the relative lack of technical information presented for the networking events (e.g. how voting/ranking took place, how word clouds were produced etc.). Could you state it explicitly whether these were held in person or online?
- More information about the attendees of the networking events and COP would be useful, especially an indicative breakdown by roles/organisations. Where do the majority of COP participants / members come from? Which groups are underrepresented apart from users of social care?

Is the work clearly and accurately presented and does it cite the current literature?

Yes

If applicable, is the statistical analysis and its interpretation appropriate?

Not applicable

Are all the source data underlying the results available to ensure full reproducibility?

No source data required

Is the study design appropriate and is the work technically sound?

Yes

Are the conclusions drawn adequately supported by the results?

Yes

Are sufficient details of methods and analysis provided to allow replication by others?

Partly

Reviewer Expertise:

social care research

I confirm that I have read this submission and believe that I have an appropriate level of expertise to confirm that it is of an acceptable scientific standard.

# Response

**Published:** 2024-06-27

**Authors:** Ferhana Hashem

**Affiliations:** CHSS, University of Kent, Canterbury, England, UK

Dear Dr Agnes Turnpenny

 We would like to thank you for your valuable comments on this manuscript. We have made the changes to the paper as you have suggested. With respect to your comment about the background and breakdown of attendees at the networking events, in order to ensure full engagement from all people taking part, we did not ask them to indicate if they were attending on behalf of an organisation or sector.

 We would like to query your comment if there were any groups apart from users of social care that were underrepresented. We would like to clarify if this question relates to inclusion of groups who are underrepresented with protected characteristics, or perhaps the question is broader and you are seeking clarification on underrepresented sector group? We did not ask people to indicate whether they belonged to a group they felt was underrepresented based on protected characteristics. Furthermore, as we did not ask people to indicate if they were attending on behalf of a specific organisation either, therefore we were unable to identify if any organisations / sector groups were underrepresented.

 We would welcome any further comments you have on the revised manuscript. Thank you.

# Reviewer report: Reviewer response for version 1

**DOI:** 10.3310/nihropenres.14603.r30313

**Published:** 2023-10-03

**Authors:** Kathryn Almack

**Affiliations:** 1 University of Hertfordshire, Hertfordshire, UK

Hashem et al’s paper explores the use of a Community of Practice (CoP) approach to facilitate research activity in social care settings. It sets out the need to improve research capacity in adult social care to develop evidence-based approaches and interventions to improve the quality of care and support. In doing so, it draws on findings from the work of the Kent Research Partnership, which is a four-year project co-led by University of Kent and Kent County Council. This is one of six capacity building social care partnerships funded by the National Institute of Health and Care Research (NIHR) Health Services and Delivery (HS&DR) programme in England.

 The paper identifies the historical (and ongoing) issues that have inhibited the development of social care research in England. These include significant pressures on social care resources which means practice rather than research is prioritised, with associated limited time and resourcing for staff to be able to access training and professional development impacting on both research capacity and capability. The authors also set out the context of funding for social care research which remains significantly lower than investments in health research.

 Against this background, the authors suggest that CoPs offer an opportunity to build forums which can focus on sharing information and insights to assist the building of knowledge and learning. The Partnership has developed two CoPs on (a) Supporting people with complex needs throughout the lifespan and (b) Enhancing, diversifying and sustaining the social care workforce. As the CoPs are in early stages of development having been launched in June 2022, the article focuses on the initial stages of their development, which offers valuable insights and learning about setting up CoPs in a social care landscape. It was also interesting to learn that ethical approvals were not required to set up the CoPs, although I would invite the authors to reflect on whether this will be revisited should the team wish to interview members of the COPs about the experience and impact of their participation in the CoPs later in the process.

 The paper describes a process of identifying and ranking research priorities to form key themes to take forward into the CoPs. To do this, the team facilitated a series of networking events ‘to engage managers and practitioners from the local authority and from the wider adult social care sector’ as well as experts by experience. It would be interesting and informative to learn more about how stakeholders were identified and to have a breakdown of stakeholders involved (for example numbers of managers, front line practitioners, commissioners, experts by experience; those from local authorities and from the voluntary ASC sector) as well as more detail about numbers and make-up of the CoPs would be good too. Are the same people in both CoPs?

 The setting up of the CoPs, including training to facilitate members using online learning resources is described in the paper - illustrative of the significant level of resourcing required to run the COPs and support ongoing engagement. The authors rightly identify an underlying concern about the sustainability of this model of creating and building research in the social care community.  I look forward to future publications exploring issues of sustainability as well as the impacts of the CoPs.

Is the work clearly and accurately presented and does it cite the current literature?

Yes

If applicable, is the statistical analysis and its interpretation appropriate?

Not applicable

Are all the source data underlying the results available to ensure full reproducibility?

No source data required

Is the study design appropriate and is the work technically sound?

Yes

Are the conclusions drawn adequately supported by the results?

Yes

Are sufficient details of methods and analysis provided to allow replication by others?

Yes

Reviewer Expertise:

Social care research; qualitiave research; communities of practice

I confirm that I have read this submission and believe that I have an appropriate level of expertise to confirm that it is of an acceptable scientific standard, however I have significant reservations, as outlined above.

# Response

**Published:** 2024-06-27

**Authors:** Ferhana Hashem

**Affiliations:** CHSS, University of Kent, Canterbury, England, UK

Dr Professor Kathryn Almack

 We would like to thank you for your thorough review of the manuscript. We have accepted the changes you have recommended. Although the priority setting exercise discussed in the paper did not require ethical approval, we have since received ethical approval for research pending involving interviews with COP members. We have stated this in the revised manuscript under the 'Consent and Ethics' section. With respect to identifying the background and breakdown of the stakeholders involved, as a networking event we wanted to ensure full engagement and did not ask attendees if they were attending on behalf of an organisation or sector.  We welcome any further feedback from a future review and would be happy to address those suggestions.

# Reviewer report: Reviewer response for version 1

**DOI:** 10.3310/nihropenres.14603.r30318

**Published:** 2023-10-03

**Authors:** Kate Hamblin

**Affiliations:** 1 University of Sheffield, Sheffield, UK

The paper provides an overview of the development of two Communities of Practice (COPs) focused on social care in the Southeast of England. The rationale for the development of the COPs is presented as facilitating the development of workforce research capacity and skills within social care, which is a sector the paper argues is under-resourced and represented in terms of research. The paper includes a detailed account of the COP process used, and insight into the early outcomes and learning. It is a useful resource to those wanting to explore the process and benefits of establishing COPs related to social care, and also other sectors.

 There are some points in the paper where being clearer around the COPs’ aims regarding ‘research capacity’ would be helpful. For example, in the plain English summary, there is reference to ‘few opportunities for the social care workforce to develop their skills and experiences to undertake their own research’ before clarifying the aims were to ‘help the social care workforce foster a culture of research and learning’. Paragraph 2 of the introduction argues COPs ‘lay the foundations for an active social care research community’ but the reader finds out about the wider programme of work on pg. 9, which is quite late in the paper. I think insight into this earlier would help the reader who in the opening sections reads about some big challenges in terms of research skills and barriers to the social care workforce undertaking research themselves, but then find capacity building sessions focused on these skills seem to come after the initial stages of the COPs and towards the end of the paper. The authors might consider an earlier framing of COPs as part of a process – including the ‘partnership’s other capacity strengthening workstreams’ – that is, moving towards empowering the social care workforce to undertake their own research.  My concern is that the sections that introduce the COPs make a strong case around the social care workforce not having the skills or capacity to conduct research themselves, and a reader could fail to appreciate the COPs were focused on developing a research culture, rather than delivering training to enable the care workforce to conduct their own research (which happens elsewhere in the programme- or could happen in the COPs in future?).

 The conclusion of the abstract also suggests knowledge exchange and dissemination as the outcomes of the COPs- again, these could be framed as part of a wider effort to create a research community in the social care sector, which in turn could foster more research developed and delivered by the social care workforce. I did also wonder whether some of the introductory paragraph that set up the ‘problem’ speak to challenges related to a lack of social care research more generally, rather than just the social care workforce’s capacity to undertake research themselves- knowledge exchange could therefore facilitate the development of new projects and practice that could help to address this wider issue.

 I do have a few other minor suggestions that may be helpful.

 Plain English Summary

- Very clear, but is there a missing word/ comma in the first line? “Social care research looks into how care and support is delivered enabling people to continue to be independent, keep their dignity and help them achieve a better quality of life”.

Abstract:

- There is an omission in the abstract in terms of explicitly naming the focus as the social care workforce, which means it doesn’t quite align with the title or main content of the paper. As a result, the ‘background’ section of the abstract doesn’t make it as clear as the plain language summary that the focus is on developing social care research and research capacity within the care sector itself, rather than facilitating social care research by external academic/ research organisations. This could very easily be amended which could facilitate the discoverability of the paper: e.g. “The landscape for supporting research in adult social care has been rather bleak, but in recent years there has been recognition of the need to foster a social care workforce research community”.
- The abstract could also perhaps include a linking sentence from the ‘problem’ statement in the ‘background’ to introducing COPs to ensure the reader understands why this approach was selected to support the development of a research community. 

Introduction

- The order of paragraphs in introduction would perhaps flow better if paragraph 2 on COPs lead into the main section of the paper, rather than in the middle of a discussion regarding the challenges and implications of a lack of workforce engagement in research.
- “Since the early 2020s, the NIHR have committed to investing in social care research capacity building by funding six adult social care partnerships, one of which is based in Southeast England involving a consortium of organisations led by the local authority and the county’s largest higher education institute (HEI).”- I wondered about a footnote here to reference other NIHR initiatives such as https://www.nihr.ac.uk/explore-nihr/funding-programmes/nihr-local-authority-academic-fellowship-programme-and-associated-opportunities.htm
- Also, in introduction- “Unlike the health sector where research income investments are substantial, generalisable, large-scale studies on social care delivery are few and far between. Social care research studies tend to be small-scale or regional in focus and hard to scale-up. The lack of controlled designs (such as randomised controlled studies (RCTs) or cluster RCTs), paucity of longitudinal research and an over-reliance on observational or cross-sectional research, further raises questions about quality of evidence”. This takes a very particular view as to the hierarchy of evidence, which feels a bit at odds with some of the discussion in the second source referenced (Rainey et al., 2016) around tensions between natural and social science approaches used in public health and social care research respectively. It also runs slightly counter to parts of the section ‘Why is it important to improve research capacities in adult social care?’ which references the contributions of people with experience of social care, who other authors argue are marginalised by traditional hierarchies of research (e.g. Glasby, J., & Beresford, P. (2006). Commentary and issues: Who knows best? Evidence-based practice and the service user contribution. Critical Social Policy, 26(1), 268-284).

Why is there low research capacity in the social care workforce?

- Might be helpful for an international audience to know how many local authorities there are to get a sense of how large a sample 70 is in the survey cited.

Collaboration process developing the COPs

- The approach to COP was explained clearly, but figure 1 and the axis of figure 3 are a little fuzzy in the pdf version of the paper.
- A little more information on the inductive-deductive coding approach (p. 6) as applied here would be interesting, if word count would allow.

Is the work clearly and accurately presented and does it cite the current literature?

Yes

If applicable, is the statistical analysis and its interpretation appropriate?

Not applicable

Are all the source data underlying the results available to ensure full reproducibility?

No source data required

Is the study design appropriate and is the work technically sound?

Yes

Are the conclusions drawn adequately supported by the results?

Yes

Are sufficient details of methods and analysis provided to allow replication by others?

Yes

Reviewer Expertise:

Adult social care, technology, ageing

I confirm that I have read this submission and believe that I have an appropriate level of expertise to confirm that it is of an acceptable scientific standard, however I have significant reservations, as outlined above.

# Response

**Published:** 2024-06-27

**Authors:** Ferhana Hashem

**Affiliations:** CHSS, University of Kent, Canterbury, England, UK

Dear Dr Kate Hamblin

 We would like to thank you for your in-depth feedback. We have amended the abstract and paper according to your suggestions. This has substantially improved the readability of the paper. We have included new figures (Figures 1 & 3) for greater image clarity of the charts. Thank you.
